# Genetic Diversity and Population Structure of Cowpea (*Vigna unguiculata* (L.) Walp.) Landraces from Portugal and Mozambique

**DOI:** 10.3390/plants12040846

**Published:** 2023-02-13

**Authors:** Joana Bagoin Guimarães, Cátia Nunes, Graça Pereira, Ana Gomes, Nascimento Nhantumbo, Paula Cabrita, José Matos, Fernanda Simões, Maria Manuela Veloso

**Affiliations:** 1 Unidade Estratégica de Investigação e Serviços de Biotecnologia e Recursos Genéticos, Instituto Nacional de Investigação Agrária e Veterinária, Instituto Público, Av. República, 2784-505 Oeiras, Portugal; 2 Divisão de Agricultura, Instituto Superior Politécnico de Manica (DivAG-ISPM), Campus de Matsinho, Vanduzi, Manica CEP 0607-01, Mozambique; 3 Centre for Ecology, Evolution and Environmental Changes, Faculdade de Ciências, Universidade de Lisboa, Campo Grande, 1749-016 Lisboa, Portugal

**Keywords:** on-farm conservation, SSR, SilicoDArT, SNP, genetic differentiation, plant genetic resources

## Abstract

Cowpea (*Vigna unguiculata* (L.) Walp.) is currently a legume crop of minor importance in Europe but a highly relevant staple crop in Africa and the second most cultivated legume in Mozambique. In Portugal and Mozambique, cowpea’s phenotypic and genetic variation has been maintained locally by farmers in some areas. We used the molecular markers SSR, SilicoDArT and SNP to analyze the genetic diversity and population structure of 97 cowpea accessions, mainly from Portugal (Southern Europe) and Mozambique (Southern Africa). As far as we know, this is the first time that the genetic variation and the relationship between cowpea landraces collected in Portugal with those originated in Mozambique is reported. Despite the shared historical past, the Portuguese landraces did not share a common genetic background with those from Mozambique, and two different gene pools were revealed. Knowledge of the genetic structure of cowpea landraces offers an opportunity for individual selection within landraces adapted to particular eco-physiological conditions and suggests the existence of a valuable gene pool for exploitation in future Portugal-PALOP (Portuguese-speaking African countries) cowpea breeding programs.

## 1. Introduction

Cowpea (*Vigna unguiculata (L.)* Walp.) is a self-pollinating diploid (2n = 22) species of the Fabaceae family, the third-largest family of plants and the second most important concerning human nutrition [1]. Cowpea is among the top five food legumes or pulses grown worldwide [2]. Its high protein content (25%, dry weight basis) is key in alleviating malnutrition and poverty, especially in Sub-Saharan Africa (SSA). In particular, in Portuguese-speaking African countries (PALOP) such as Angola, cowpea is an important staple crop [3], and in Mozambique, it is, at present, the most cultivated legume [4,5].

The worldwide importance of legumes is also linked with their ability to fix atmospheric nitrogen, contributing to the structure of ecosystems and sustainable agriculture [6]. Some legumes fix nitrogen better than others, with cowpea in an average position, fixing more than common bean and less than faba bean and soybean [7]. Together with its ability to withstand poor soils and partial shadow, cowpea is often used in intercropping with cereals [8] and rotation systems. Furthermore, cowpea is particularly drought-tolerant, a crucial ability in its dry area of origin [9] and in areas affected by climate change, such as the Mediterranean basin [10]. As cowpea is grown for food (leaves, pods and seeds), fodder, green manure and as a cover crop [11], recent development efforts focused on dual-purpose varieties with both high grain and fodder yields [12].

The center of diversity for cultivated cowpea is reported to be in West and Central Africa. The oldest evidence that cowpea existed in West Africa was obtained from carbon dating specimens from the Kimtampo rock shelter in Central Ghana [13]. The center of origin of wild cowpea is Southern Africa and the accepted progenitor of cowpea is *Vigna unguiculata ssp. dekindtiana* (Harms) Verdc., widely distributed across Africa [14]. Huynh et al. [15], using single-nucleotide polymorphism (SNP) markers, concluded that cowpea originated from two divergent domestication processes because two gene pools were identified, one in West Africa and another in East Africa.

The narrow genetic base of improved cowpea varieties can be attributed to its self-pollinating characteristics [16]. However, there are robust cowpea molecular markers that allow discrimination among germplasm accessions and variety identification, including SSR [16,17] and SNP markers under the Kompetitive Allele-Specific PCR (KASP) [18]. Genetic marker analysis through DArT is also a robust system that requires minimal DNA samples and provides comprehensive genome coverage [19]. This technology, based on microarray hybridizations, can detect DNA variation, and the presence versus absence of individual fragments, without the need for sequence information. DArT analysis was already applied to the germplasm characterization of barley [20], strawberry [21], macadamia [22] and, to our knowledge, very recently to cowpea from Ethiopia [23] and Togo [24].

In Portugal, there is a detailed morphological description of the landraces grown in the country in the decade of the 1950s, performed by Castillo [25]. Later, Reis [26] studied 24 accessions collected in farmers’ fields at distinct Portuguese ecological regions using isoenzymes. Stoilova and Pereira [27] studied the variation in the quantitative and qualitative traits of Bulgarian and Portuguese landraces and confirmed the existence of large morphological variability in the national accessions. It was found that high values concerning the number of pods and seeds per plant, the most important components of yield, were present in one Portuguese landrace. More recently, Carvalho et al. [28] compared Iberian Peninsula with worldwide cowpea accessions using SNP markers. The authors put forward possible dispersion routes of cultivated cowpea. Nowadays, a limited number of cowpea local populations are still cultivated in Portugal, primarily for human consumption of the grains. Despite the previous studies performed on *V. unguiculata* of Portugal, knowledge is lacking on the Portuguese landraces, which were only partially represented. Characterization and knowledge of the existing levels of diversity within and among these still-cultivated landraces are necessary to establish suitable on-farm conservation strategies and as a first step to introduce these populations in future breeding programs. In Mozambique, despite it being considered a diversity hot-spot for cowpea [4] and cowpea being a key agronomic culture, scarce breeding programs have failed to deliver farmers improved and adapted varieties. Instead, farmers hold landraces empirically selected over the years with very much unknown diversity and agronomic potential. Recently, efforts have been made to preserve and study this diversity [4,29] in an attempt to promote breeding programs, but it still requires further research.

In this work, we aimed to examine the genetic diversity and structure of Portuguese and Mozambican cowpea landraces, mainly to explore the potential relatedness in existing cowpea landraces in these two Portuguese-speaking countries, where such research has not been conducted yet, and to assess the pertinence of cross-country breeding programs. This knowledge will increase the efficiency in the sustainable conservation and utilization of Portuguese and Mozambican *V. unguiculata* genetic resources. The Portuguese accessions used were collected in farmers’ fields since the decade of the 1980s and were stored at “Banco Português de Germoplasma Vegetal”, with some populations still being cultivated by farmers. There are 199 accessions stored in the Portuguese bank, but for our study, only the most relevant and representative accessions of the different growing areas were chosen. The Mozambican cowpea landraces originated from two gene banks. The first was the Mozambican Germplasm Bank kept at the “Instituto de Investigação Agrária de Moçambique (IIAM)”, the oldest collection in the country. The second source was the newly established “Instituto Superior Politécnico de Manica Gene Bank” in Central Mozambique, which has the highest and most recent collection of cowpea germplasms from Central Mozambique. The accessions used in this study are relevant and distributed through the country. However, they do not yet represent all the existing genetic variability of Mozambican cowpea because collection missions are still ongoing.

## 2. Results

### 2.1. Overall Genetic Diversity

To compare the genetic variation of *V. unguiculata* populations from Portugal and Mozambique, 34 and 52 accessions, respectively, were analyzed using SSR, SilicoDArT and SNP markers. In the SSR analysis, there were additionally 5 accessions from Angola, 3 from Cape Verde, 1 from Spain, 1 from Greece and 1 from Nigeria (Table S1). For SilicoDArT and SNP markers, in addition to Portuguese and Mozambican accessions, we used only 4 of the 5 accessions from Angola, 1 of 3 accessions from Cape Verde and the same from Nigeria. Three *V. unguiculata ssp. sesquipedalis* (L) Verdc. accessions, from Portugal, Spain and Cape Verde, were used as an out-group. The accession IT-97K-499-35 from Nigeria was also included in the study because it is the reference genome for cowpea.

#### 2.1.1. SSR Markers

Twelve SSR loci amplified a total of 183 alleles, with an average of 15.25 alleles, ranging from 4 (CLM0634) to 27 (VuUGM71), and with an average effective number of alleles (Ne) of 2.61. The Polymorphism Information Content (PIC) ranged from 0.354 (VM5) to 0.892 (VuUGM33), indicating that the used loci displayed a high level of variability and are useful diversity indicators. The locus VuUGM71 displayed the highest values of Ne and He, while the locus CLM0634 displayed the lowest values. The mean observed heterozygosity (Ho) was 0.325 and the mean expected heterozygosity (He) was 0.461 (Table 1).

#### 2.1.2. SilicoDArT and SNP Markers

A total of 61,221 polymorphic SilicoDArT (Table S2) markers were generated, of which 9251 were aligned with the marker sequences obtained from bacteria (NCBI) and 8361 from expressed sequence tags (EST) of several plant species. All the markers (61221) showed ≥95% reproducibility. All the identified SilicoDArT markers had a call rate value ≥ 75% (Figure S1) with an average value of 94.45% (Table S2). Low-frequency markers can affect the statistical analysis. As such, 25,961 markers with extremely low one ratio (<0.05) were not considered in the analysis.

**Figure 1:**
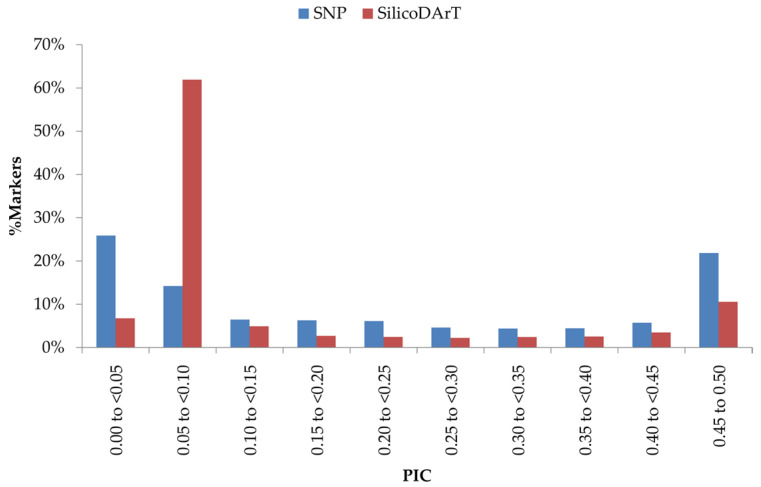
Distribution of PIC values of SilicoDArT and SNP markers used for genomics studies in cowpea.

In total, 31,420 SilicoDArT markers were selected for the study. Among these informative markers, around 9% were observed in PIC class 0.45 to 0.5 and 50% in the 0.05 to 0.1 class (Figure 1). The PIC values of the remaining markers were distributed almost equally (2–5%) across the rest of the marker groups. The median (0.05) was located close to the average PIC value of 0.14 (Table S3).

A total of 38,889 SNP markers (Table S4) were identified having an average of 99 % reproducibility and 86% call rate. Around 98% of SNP markers had ≥95% reproducibility, of which 32,903 were found to be 100% reproducible (Figure S1). The call rate exhibited variance, ranging from 20% to 100%. Around 44% of SNP markers displayed a <75% call rate (Figure S1) and were not considered for this study. For the remaining markers, 26,085 showed a >90% call rate; 19,538 of all the identified markers had >0.5 average one ratio. Considering all the quality parameters, 11,050 SNP markers were used for subsequent analysis. These markers were determined to be highly informative, with an average PIC value of 0.17 and a median of 0.06 (Table S3). Around 42% of markers were in the lowest PIC value range (0–0.05) and 15% in the highest PIC value range (0.45 to 0.50) (Figure 1). The remaining PIC value groups exhibited an approximately similar marker frequency value, ranging from 3 to 15% each.

All possible SNP types were found in our study (Table S5). The SNPs G/A, C/T, T/C, A/G and T/A were the most common in this set of cowpea accessions (15.1, 14.2, 13.5, 12.7 and 8.5 percent, respectively). SNPs were randomly distributed across cowpea genomes and their chromosome allocation was as follows: Chromosome 1: 6.1%; Chromosome 2: 6.7%; Chromosome 3: 12.8%; Chromosome 4: 9.9%; Chromosome 5: 8.5%; Chromosome 6: 7.7%; Chromosome 7: 11.5%; Chromosome 8: 7.2%; Chromosome 9: 6.2%; Chromosome 10: 7.6% and Chromosome 11: 8.4% (Table S4).

### 2.2. Diversity and Genetic Relationships between Accessions

#### 2.2.1. SSR

**Figure 2:**
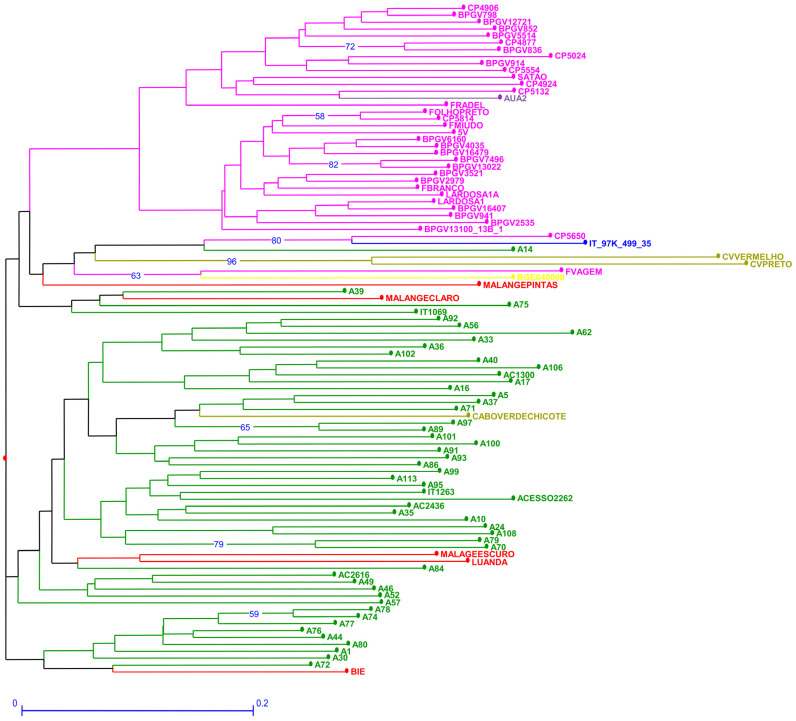
Neighbor joining dendrogram of cowpea genotypes, identified by the twelve SSR markers. Numbers associated with branches indicate bootstrap values (BS) based on 20,000 replications. Only BS values above 50 are shown. Branch lines represent individual accessions grouped by country (red: Angola, olive green: Cape Verde; yellow: Spain; purple: Greece; green: Mozambique; blue: Nigeria and pink: Portugal).

The genetic dissimilarity among accessions estimated through SSR markers ranged between 0.125 and 1 (Table S6). The out-group (*ssp. sesquipedalis*) was not particularly separated from the remaining accessions (Figure 2). Portuguese F. vagem and Spanish BGE040000 scored very close (Figure 2), while CV-Chicote from Cape Verde was among the Mozambican accessions with no particular separation. Lardosa1A, an important traditional Portuguese landrace, scored among all Portuguese accessions. The same happened for another traditional, still cultivated landrace, Satão. Interestingly, Portuguese CP5650, recently collected, is positioned in the same branch as the reference accession IT_97K_499_35 from Nigeria, which scored between Portuguese and Mozambican accessions. CV vermelho and CV preto from Cape Verde were the most distant accessions.

#### 2.2.2. SilicoDArT

**Figure 3:**
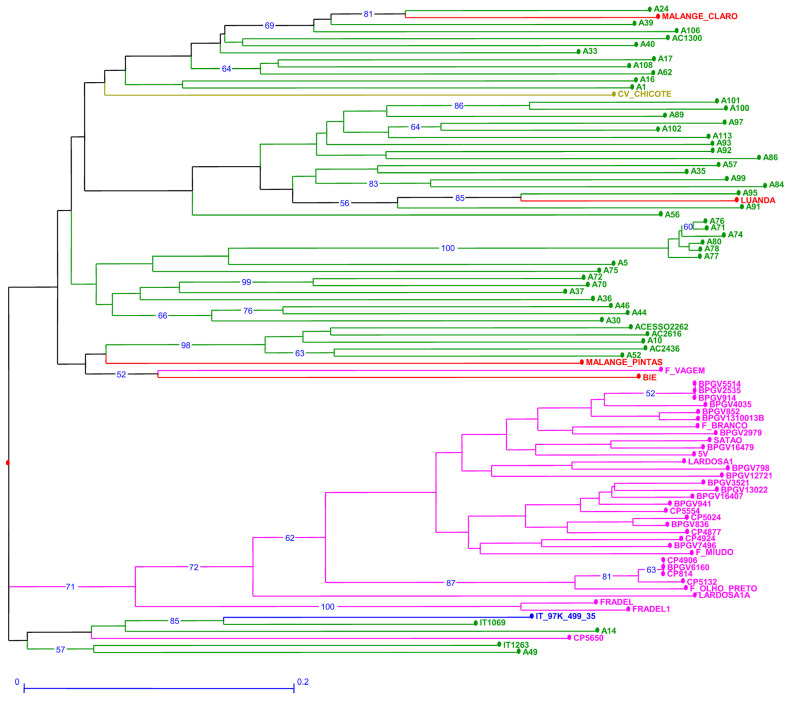
Neighbor joining dendrogram of cowpea genotypes, identified by SilicoDArT markers. Numbers associated with branches indicate bootstrap values (BS) based on 20,000 replications. Only BS values above 50 are shown. Branch lines represent individual accessions grouped by country (red: Angola, olive green: Cape Verde; green: Mozambique; blue: Nigeria and pink: Portugal).

The genetic dissimilarity among accessions estimated through SilicoDArT markers ranged from 0 to 1 (Table S7). CV-Chicote (*ssp. sesquipedalis*) from Cape Verde was, as expected, isolated in a branch (Figure 3). However, the other out-group sample, F. vagem from Portugal, scored far from CV-Chicote and close to two accessions from Angola (Figure 3) in a branch shared by Mozambican accessions. Lardosa1A, an important Portuguese landrace, scored among Portuguese samples but on an isolated branch. Only Frade scored even further. Satão, however, is clearly similar to most Portuguese accessions. An additional branch groups the reference accession with the breeding lines IT1069 and IT1263, Portuguese CP560 and A14 and A49 from Mozambique. SilicoDArT did not discriminate between BPGV2535, BPGV 5514 and BPGV914 or between CP4906, CP5814 and BPGV6160.

#### 2.2.3. SNP

**Figure 4:**
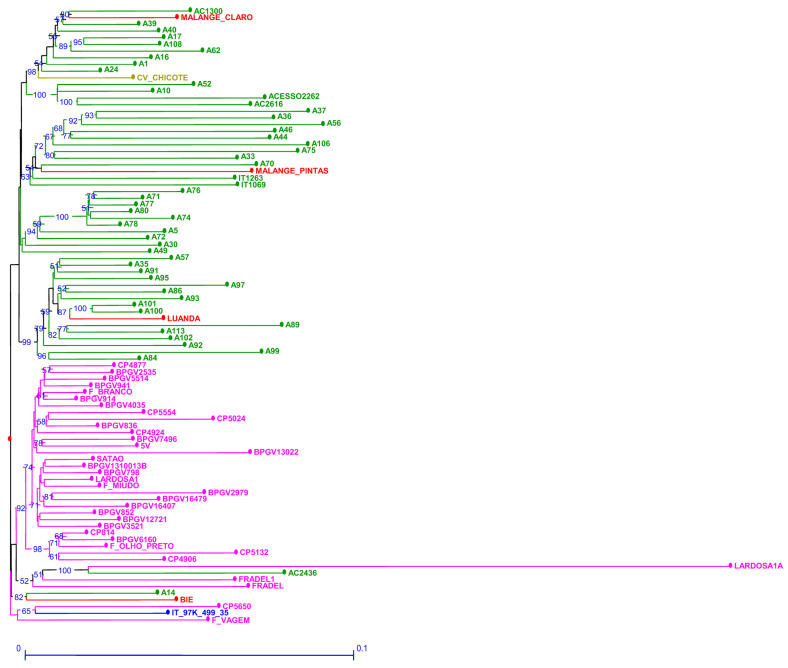
Neighbor joining dendrogram of cowpea genotypes, identified by SNP markers. Numbers associated with branches indicate bootstrap values (BS) based on 20,000 replications. Only BS values above 50 are shown. Branch lines represent individual accessions grouped by country (red: Angola, olive green: Cape Verde; green: Mozambique; blue: Nigeria and pink: Portugal).

The genetic dissimilarity among accessions estimated through SNP markers ranged from 0.023 to 0.309 (Table S8). SNP markers were able to discriminate all samples. Lardosa1A (traditional Portuguese landrace) is the accession with the higher genetic dissimilarity when compared to all other accessions grouping together on the same branch with AC2436 (Figure 4). In relation to the out-group “*ssp. sesquipedalis*”, CV-Chicote from Cape Verde and Portuguese F. vagem scored quite far from each other but on their own branches. An additional branch groups the reference accession with Portuguese CP5650 and F. vagem.

In general, the three marker types, SSR, SilicoDArT and SNP, clearly separated samples from Mozambique and Portugal; both accessions “ssp. *sesquipedalis*” scored quite distantly from each other and Lardosa 1A was identified as singular among the Portuguese accessions.

### 2.3. Differentiation of Populations: Bayesian Approach and PCA Analysis

For all markers, based on the results of the STRUCTURE analysis, it was considered that an accession with a score higher than 0.80 was pure, while, if with a lower score, it was considered admixed.

#### 2.3.1. SSR

**Figure 5:**
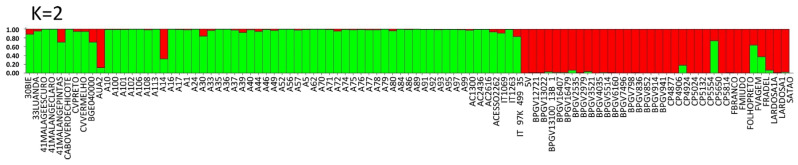
Population STRUCTURE of 97 cowpea accessions using SSR marker data as estimated using the model-based Bayesian algorithm implemented in the STRUCTURE program. Proportion of assignment of individuals to K = 2 subpopulation groups. Each accession is represented by a vertical line. Vertical lines with only one color (higher than 80%) were considered pure accessions.

The Bayesian approach indicated that the most likely number of genetic clusters was K = 2 (ΔK = 2) (Figure S2). Based on the STRUCTURE analyses, the two groups (gene pools) assigned at K = 2 correspond to landraces from Europe (red color) and from PALOP (green color) (Figure 5). Exceptions to this observation were the admixed samples, which belong to both groups (with less than 80% of each color), including BGE040000 (Spain) and F. vagem, which are both *V. unguiculata ssp. sesquipedalis*, one Portuguese landrace (CP5650) and the Portuguese commercial variety (Fradel). The accession A14 from Mozambique is also admixed (Table S9).

**Figure 6:**
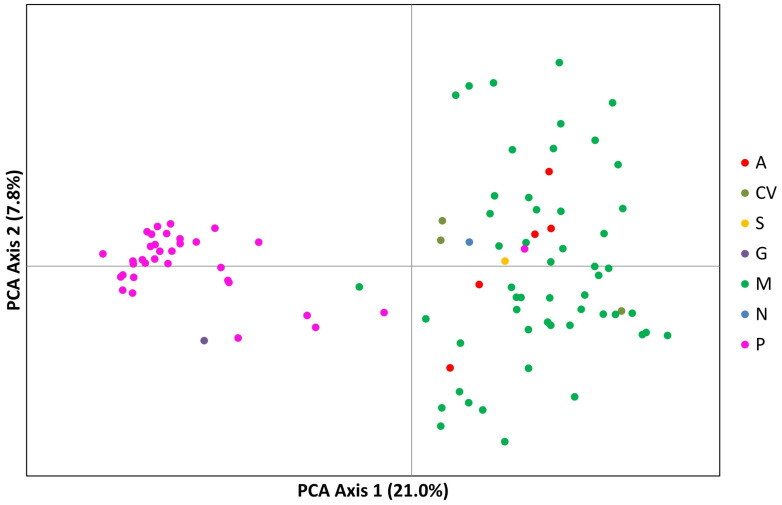
Principal component analysis (PCA) of SSR markers showing allelic variation among 97 cowpea accessions. Dots represent individual accessions grouped by country (red: Angola, olive green: Cape Verde; yellow: Spain; purple: Greece; green: Mozambique; blue: Nigeria and pink: Portugal).

A PCA analysis of the populations explained 28.76% of the variation in the first two axes, 21.01% in the first component and 7.75% in the second component. The Portuguese landraces are on the left side of the graph, while the accessions from Mozambique are on the right side of the graph. The Portuguese landraces CP5650, Lardosa 1A, F. vagem and the commercial variety Fradel did not group with the remaining Portuguese samples. The samples from Angola and Cape Verde are scattered within the Mozambican cloud (Figure 6).

Wright’s FST and Slatkin´s RST were used as a measure of the extent of the genetic differentiation among the populations. These parameters are useful differentiation estimators, commonly used to describe population structuring through SSR markers [30,31]. When used to compare cowpea populations from Portugal and Mozambique, some degree of differentiation (FST = 0.220; RST = 0.194) was observed.

#### 2.3.2. SilicoDArT

The values of ΔK estimated from SilicoDArT markers peaked at K = 2 and K = 4 (Figure S2). At K = 2, the cluster inference indicates two major populations (gene pools), Portugal (red color) and PALOP (green color).

**Figure 7:**
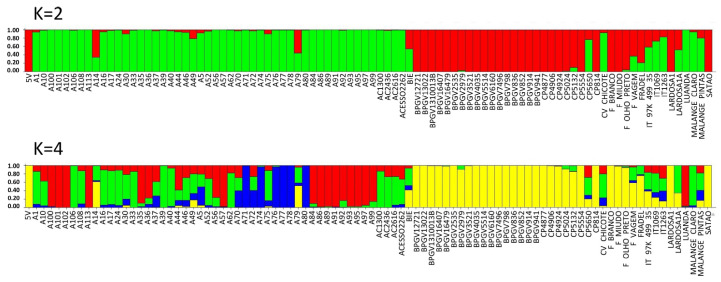
Population STRUCTURE of 92 cowpea accessions using SilicoDArT marker data as estimated using the model-based Bayesian algorithm implemented in the STRUCTURE program. Proportion of assignment of individuals to K = 2 and K = 4 subpopulation groups. Each accession is represented by a vertical line. Vertical lines with only one color (higher than 80%) were considered pure accessions.

Applying K = 4, four subpopulations were observed: one from Portugal (yellow) and three from PALOP landraces (red, green and blue). The majority of the Portuguese landraces were pure accessions (88%, with a score higher than 0.80). Exceptions were the landraces CP5650 (admixed of red, green and yellow), Lardosa 1A (green and yellow), F. vagem, which is *V. unguiculata ssp. sesquipedalis* (all colours), and the commercial variety Fradel (mainly green and yellow) (Figure 7, Table S10). The breeding lines, reference cultivar IT-97K-499-35 and the Cape Verde accession are also admixed, included in all subpopulations (Figure 7, Table S10). From Angola, there are admixed accessions included in all subpopulations, and also two pure accessions, one from the green subpopulation (Malange Claro) and the other red (Luanda).

There are 58% of Mozambican landraces that are pure, spread across three subpopulations (29% red, 17% green and 11% blue). All the other landraces were admixed. Several landraces from the agroecological region R7, described by Gomes et al. [4] (A71, A74, A76, A77 and A80), have no admixture and are clustered in subpopulation blue (Figure 7, Table S10).

**Figure 8:**
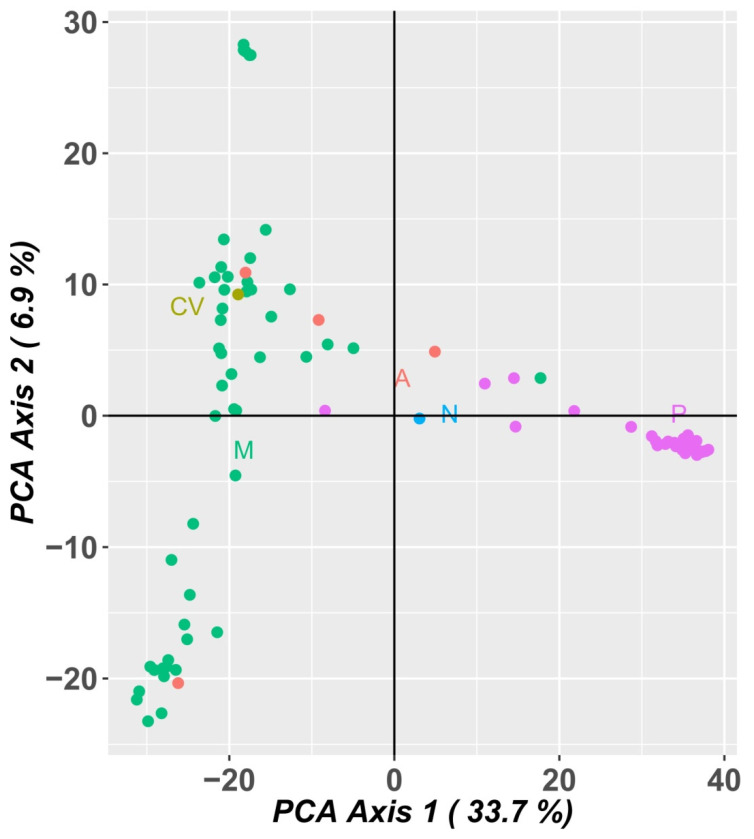
Principal component analysis (PCA) of SilicoDArT markers showing allelic variation among 92 cowpea accessions. Dots represent individual accessions grouped by country (green: Mozambique; olive green: Cape Verde; orange: Angola, blue: Nigeria and pink: Portugal).

A PCA analysis of the populations explained 40.6% of the variation in the first two axes, 33.7% in the first component and 6.9% in the second component (Figure 8).

The Portuguese landraces are on the right side of the graph, while the accessions from Mozambique are on the left side of the graph. The Portuguese landraces CP5650 and Lardosa 1A and the commercial variety Fradel and F. vagem (*V. unguiculata ssp. sesquipedalis*) did not group with the remaining Portuguese samples.

The samples from Mozambique may be grouped into three different clusters. The landraces A76, A78 and A80 (upper left side of the graph), sampled at the agroecological zone R7, described by Gomes et al. [4], are the most distant from all the other samples from Mozambique.

#### 2.3.3. SNP

Considering population structure analyses using SNP markers, the Evanno’s ΔK peaked at K = 3 and K = 5 (Figure S2). At K = 3, the cluster inference indicates three populations (gene pools): two from Portugal (blue and red) and one from PALOP (green). Applying K = 5, two pure populations from Portugal (blue and pink), two pure populations from PALOP (red and yellow) and a green gene pool among admixed samples were observed. The blue subpopulation included 74% pure Portuguese landraces. The Portuguese admixed landraces were CP5650, CP5132, CP5024, CP4906, BPGV 2979, F. vagem (out-group *ssp. sesquipedalis*) and the commercial variety Fradel. With the exception of BPGV 2979, all the other admixed landraces were collected after 2004. Landrace Lardosa 1A stands out as a unique population with no admixture (pink).

**Figure 9:**
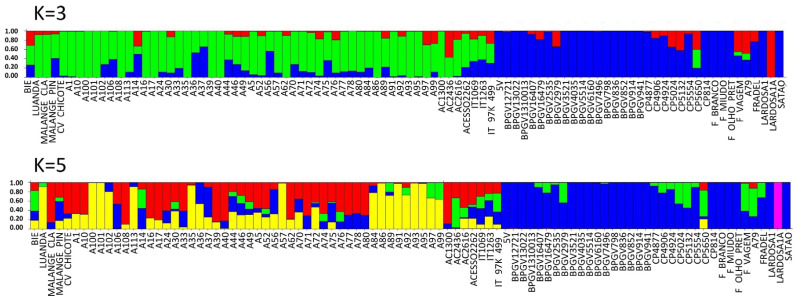
Population STRUCTURE of 92 cowpea accessions using SNP marker data as estimated using the model-based Bayesian algorithm implemented in the STRUCTURE program. Proportion of assignment of individuals to three and five population groups. Each accession is represented by a vertical line. The distribution of the accessions to different populations is indicated by the color code. In the blue right side are all the Portuguese accessions and on the other side are the PALOP samples. Vertical lines with only one color (higher than 80%) were considered pure accessions.

Most Mozambican pure accessions clustered in subpopulations that were yellow (A100, A101, A102, A113, A35, A57, A86, A91, A93 and A95) and red (A108, A17, A39, A40 and AC1300), 19% and 9.6%, respectively, with all others being admixed (Figure 9).

Two landraces of Angola were pure (Luanda (yellow) and Malange Claro (red)). The Cape Verde landrace (out-group *ssp. sesquipedalis*) was admixed (Figure 9; Table S11).

As for SilicoDArT markers, a higher degree of admixture (Figure 7, Figure 9, Tables S10 and S11) was found for the landraces from Mozambique compared to those from Portugal.

**Figure 10:**
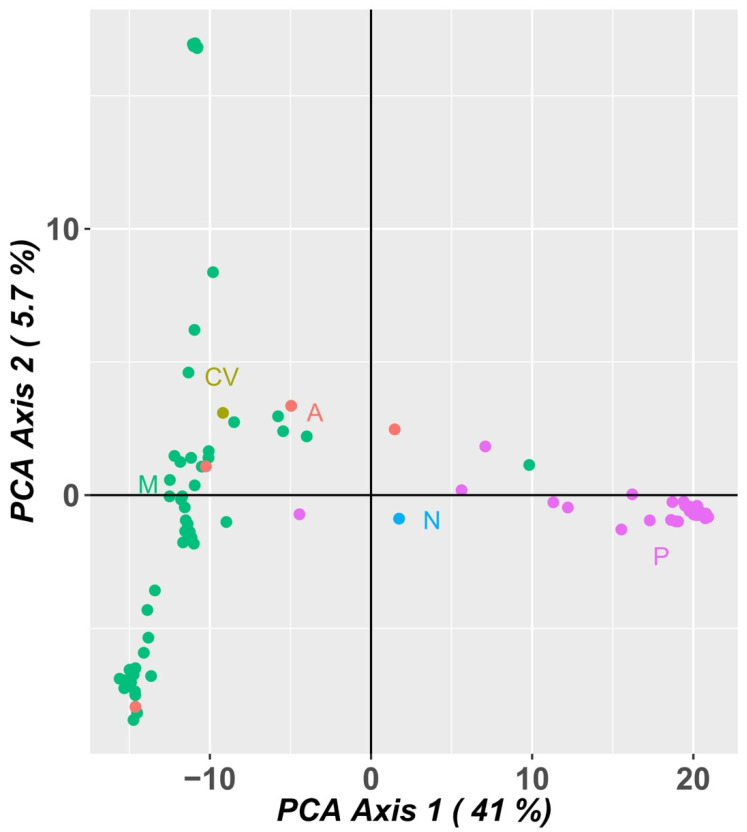
Principal component analysis (PCA) of SNP markers showing allelic variation among 92 cowpea accessions. Dots represent individual accessions grouped by country (green: Mozambique; olive green: Cape Verde; orange: Angola, blue: Nigeria and pink: Portugal).

A PCA analysis of the population using SNP marker data (Figure 10) explained 46.7% of the variation in the first two axes, 41.0% in the first component and 5.7% in the second component. The Portuguese landraces are on the right side of the graph, while the accessions from PALOP (Mozambique, Angola and Cape Verde) are on the left side of the graph. The Portuguese landraces CP5650 and Lardosa 1A and the commercial variety Fradel did not group with the remaining Portuguese samples.

The samples from Mozambique may be grouped into three different clusters. The landraces A76, A78 and A80 (upper left side of the graph), from the agroecological zone R7, described by Gomes et al. [4], are grouped and are the most distant from all the other samples from Mozambique.

The PCA analysis using SNP marker data (Figure 10) was very consistent with the PCA analysis using SilicoDArT markers, distributing the accessions in a similar way.

### 2.4. Comparison of Marker Systems

The application of different marker systems to the same gene pool stands as an opportunity to compare the performance of those marker types in diversity studies. The difference in results is a consequence of the nature of the markers and the analysis methodology.

**Figure 11:**
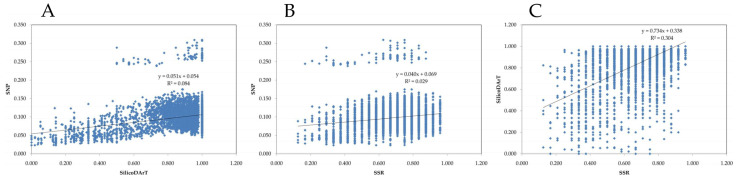
Mantel correlation test between SilicoDArT and SNP markers (**A**), SSR and SNP markers (**B**) and SSR and SilicoDArT markers (**C**).

In our study, all marker types provided similar evidence about the germplasm collection but with some differences worth noting: overall, the three marker systems divided accessions into two main subpopulations, separating Portuguese accessions from Mozambican accessions. However, SNP markers were able to discriminate a third subpopulation with one single accession, Lardosa 1A (Figure 11). When pushing Evanno’s ΔK further, SilicoDArT markers were able to divide the accessions into four subpopulations and SNPs into five, admitting further separation of the accessions from Mozambique, again with only SNPs recognizing Lardosa 1A as a separate subpopulation. SSRs were not able to provide this differentiation, possibly a consequence of the relatively small number of markers used. When considering isolated accessions, there were differences in distance among the three methods, but, again, the overall conclusions are the same, with Lardosa1A being isolated within the Portuguese subpopulation in all marker systems and the out-group (ssp. *sesquipedalis*) not being discriminated, as expected, by any of the markers. To assess the correlation between the used marker systems, we have applied the Mantel test. Despite low r values, a significant correlation (*p* < 0.001) was found for the three comparisons (Figure 11).

## 3. Discussion

Cowpea is currently a legume crop of minor importance in Europe. However, its relevance is likely to increase in the near future, considering the dual combination of its great resistance to drought and the predicted scenario of increased temperatures and decreased rainfall in Europe. In the PALOP, however, as in most of Sub-Saharan Africa, cowpea is key for rural families. Landraces are traditional crop varieties maintained by farmers without any formal improvement. Portugal and Mozambique are still rich in cowpea landrace diversity, maintained on-farm and ex situ.

As a consequence of the observed impacts of climate change, already very pronounced in the last few years, it is crucial to promote global food systems based on family farming using sustainable production methods that preserve biodiversity and healthy diets. A methodology for territorial intervention has been defined by the International Forum Relevant Territories for a Sustainable Food Systems [32], which, in several parts of the world, established Eco-Regions. An Eco-Region aims to avoid the abandonment of rural areas and the loss of biodiversity and ancestral food knowledge [32].

Although some cowpea genetic erosion has been observed in Portugal during the last few decades, due to the reduction in small-scale farming, there are two important growing regions, the São Pedro do Sul and Lardosa (Idanha-a-Nova) Eco-Regions, where cowpea landraces are still preserved on-farm (Table S1).

Considering Mozambique, Eco-Regions are not yet established. However, in 2019, the second Eco-Regions World Congress within the context of the International Forum on Relevant Territories for Sustainable Food Systems decided to implement an integrated methodology to promote sustainable food systems within the frameworks of Community of Portuguese Language Countries (CPLP) Food Security and Nutrition, the Strategy of the Sustainable Development Goals and the UN Decade of Family Farming [33]. The implementation of these frameworks will be very important for the on-farm preservation of cowpea in some relevant Mozambique regions—for example, the Manica region. In this region, the crop appears to be structured as a meta-population, where substantial differentiation is maintained at the subpopulation level. Similar results were also observed in Italy by Tosti and Negri [34] when studying three cowpea landraces collected at the Umbria region.

To achieve effective conservation and enhance the use of cowpea germplasm, there is a need for a detailed characterization of the existing diversity. The genetic structure of cowpea populations is highly determined by its mating system, which is characterized by high self-pollination and, so, the cowpea crop is a mixture of a number of distinct homozygous lines [35].

In this work, we present new insights into the diversity of cowpea landraces from Portugal (Europe) and Mozambique (Southern Africa). As far as we know, this is the first time that cowpea landraces collected in Portugal are compared with those originated in Mozambique using SSR, SilicoDArT and SNP markers.

FST and RST are useful differentiation estimators, commonly used to describe population structuring [30,31]. According to the standards of Del Carpio et al. [36], some genetic differentiation was found when comparing cowpea landraces from Portugal and Mozambique when using SSRs. Previous work, using SNPs [15,37], also separated Europe from South African countries, including Mozambique.

According to the three genetic markers used in the study, at least 80% of the Portuguese landraces are pure and they are grouped in a main cluster. Lardosa 1 and Lardosa 1A are landraces grown on-farm in the same region, although only sold at local markets, and have great economic importance for traditional farmers. They are both pure landraces but differ morphologically from each other. The seed of Lardosa 1 has an ovoid form and the hilum is green, and Lardosa 1A usually called “rice bean” and has a globose form and the hilum is brown. Lardosa 1 is grouped in the main Portuguese cluster but Lardosa 1A is not. Physiological studies in Portuguese landraces showed that Lardosa 1 was able to maintain tissue hydration and water use efficiency under the imposed water deficit, which translated into a higher number of grains per plant than in other accessions, indicating adaptation to the Mediterranean climate and conveying the importance of landrace preservation [9].

CP5650 is not a pure landrace (admixed with three subpopulations), is quite distant from all the other Portuguese samples in the three PCAs and is closer to the Mozambican landraces. It should be noted that the cowpea pure landraces are mostly those stored at “Banco Português de Germoplasma Vegetal” (collected before 2000), and also Lardosa 1, Lardosa 1A and Sátão, which are still on-farm cultivated. The CP5650 landrace was only recently collected (2014), which may suggest that it was brought from another geographical region.

When analyzing the Mozambique accessions, we observed that, using SilicoDArT or SNP markers, only 50% or 27% are pure landraces. Thus, a high level of admixture was observed for these accessions, as Gomes et al. [4] previously noted when using SSR markers, suggesting the occurrence of gene flow between landraces. In the present study, SSR markers did not allow the clustering of landraces according to their geographical origin. However, SilicoDArT and SNP clustered landraces from the north of Manica (Tambara, R6 agroecological region) and Zambezia (landraces A78 and A80, R7 agroecological region). A14, which is not a pure landrace (admixed with two subpopulations), is quite distant from all the other Mozambique samples in the different scatter plots. There is the possibility that A14 had been introduced in Mozambique from Malawi, as there are informal exchanges of plant material along both countries’ borders.

Briefly, population structure analysis (using SSR, SNP and SilicoDArT) revealed two different gene pools, corresponding to Portugal (Southern Europe) and Mozambique (Southern Africa). Portuguese landraces did not share a common genetic background with those from Mozambique, confirming previous results. The genetic architecture of cowpea landraces from South Europe and Africa was studied by Huynh et al. [15] and Xiong et al. [37], who concluded that the landraces from Southern Europe were more related to those of West and Central Africa than to those of Southern Africa, namely Mozambique. Cowpea has been known in Southern Europe since the Roman times, so the crop could have been introduced through the Middle East [38], which reinforces our results.

The present knowledge of the genetic structure of cowpea landraces gives an opportunity for individual selection within landraces adapted to particular eco-physiological conditions. In particular, the assignment of molecular markers for these accessions, potentially linked to abiotic and biotic stress resistance, nutritional richness and a symbiotic capacity for nitrogen fixation, will allow the design of a specific SNP marker panel to be used in molecular-assisted breeding.

## 4. Materials and Methods

### 4.1. Plant Material

**Figure 12:**
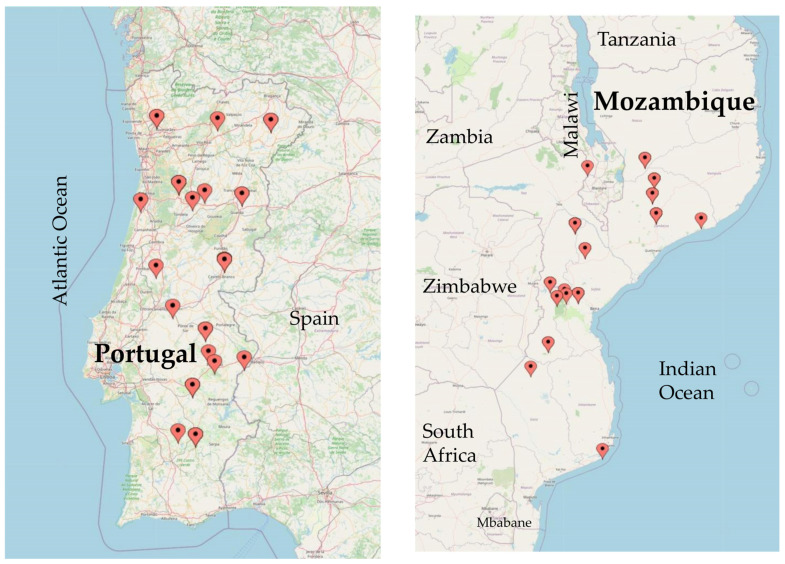
Locations of Portuguese and Mozambican accessions used in this study (https://www.mapcustomizer.com/, accessed on 9 February 2023).

A total of 97 *V. unguiculata* accessions of multiple origin were used for genetic diversity and population structure analyses by SSR: 34 from Portugal, 52 from Mozambique, 5 from Angola, 3 from Cape Verde, 1 from Spain, 1 from Greece and 1 from Nigeria (Table S1). For SilicoDart and SNP markers, in addition to the same Portuguese and Mozambican accessions (Figure 12), we used only 4 accessions from Angola, 1 from Cape Verde and 1 from Nigeria. The Portuguese sample Fradel is a commercial variety, obtained at the Portuguese Plant Breeding Station, Elvas. The remaining Portuguese samples were obtained from Banco Português de Germoplasma Vegetal (BPGV) (17 landraces), from INIAV-Elvas (8 landraces) and from farmers´ fields (8 landraces). The Spanish accession was provided by Centro de Recursos Fitogenéticos—Instituto Nacional de Investigación y Tecnologia Agraria y Alimentaria (INIA), and the Greek accession was provided by the Agricultural University of Athens, Greece. Four of the accessions from Mozambique were obtained from Banco de Germoplasma Nacional, kept at the Instituto de Investigação Agronomica de Moçambique (IIAM); 52 were obtained from Banco de Germoplasma do Instituto Superior Politécnico de Manica, and, additionally, two widely used commercial cultivars were used, IT1069 and IT1263, released by the Mozambican Institute of Agricultural Research (IIAM) and bred through a partnership with the International Institute of Tropical Agriculture (IITA) in Nigeria. IT-97K-499-35 (reference accession for cowpea) was obtained from IITA.

### 4.2. DNA Extraction, PCR Amplification and Fragment Sizing

DNA was isolated from young leaves using the innuPREP Plant DNA Kit (Analytik Jena AG, Berlin, Germany), according to the manufacturer´s protocol. DNA quality and concentration were visually checked on 0.8% agarose gel. The DNA concentration was also estimated using a NanoDrop ND2000 spectrophotometer (Thermo Scientific, Waltham, MA, USA). All accessions were genotyped twice to use as technical replicates.

### 4.3. Genotyping Cowpea Accessions Using SSR Markers

Cowpea genotyping was based on 12 SSR loci, which were developed by Xu et al. [39], Gupta and Gopalakrishna [40] and by Li et al. [16]. Primer sequences and respective labeling are provided in Table S12.

The multiplexed PCR amplification was performed with three SSR loci using the QIAGEN Multiplex PCR Kit in a final volume of 10 µL, according to the manufacturer’s instructions, in a My Cycler Biorad thermocycler.

Subsequently, 1.0 µL of the PCR mixture was added to 24 µL formamide and 0.5 µL fragment size standard labeled with WellRED dye D1 (DNA size standard kit, 400, Beckman Coulter). Capillary electrophoresis was performed to separate the PCR products using the CEQ 8000 Genetic Analysis System (Beckman Coulter Inc., Brea, CA, USA). Data analysis was performed using the CEQ 8000 Fragment Analysis software, version 9.0, according to the manufacturer´s recommendations (Beckman Coulter Inc., Brea, CA, USA). Sizes of fragments were automatically calculated using the CEQ 8000 Genetic Analysis System.

### 4.4. Genotyping Cowpea Accessions Using SilicoDArT and SNP Markers

The high-throughput DArTseq technology used to genotype cowpea accessions followed the standard procedures [20] and was performed using the service from DArT Pty Ltd. (Canberra, Australia).

Next-generation sequencing technology was implemented using HiSeq2000 (Illumina, San Diego, CA, USA) to detect SNPs and SilicoDArT markers.

### 4.5. Data Analysis

GenAlEx 6.503 [41] was used to assess the genetic diversity, measured as the number of alleles per locus (Na), observed and expected heterozygosity (Ho and He) and to calculate the pairwise standard genetic distances and the standard FST (via frequency) values. The genetic distance between each pair of individuals was calculated following Nei and Li [42]. The neighbor joining algorithm, as implemented in the DARwin software package version 6.0.12 [43], was based on a dissimilarity matrix and the reliability of the tree topology was assessed via bootstrapping over 20,000 replicates. Regarding the principal component analysis (PCA), the distance matrix was calculated following Peakall and Smouse [41] and was used to assess the diversity of all accessions. The consistency of the SilicoDArT, SNP and SSR-based distance matrixes was measured using Mantel’s test.

The differentiation between the populations was estimated using Wright´s FST and Slatkin´s RST. FST results were interpreted following Del Carpio et al. [36], where 0 indicates no differentiation between populations and a value of 1 indicates complete differentiation. Populations were considered to have great differentiation when FST values ranged between 0.15 and 0.25.

Micro checker software v2.2.3 [44] was used for the detection of null alleles, stuttering and allele dropout.

The level of genetic stratification among the studied germplasm was assessed using STRUCTURE v.2.3.4 software [45]. The analysis was performed considering both the admixture model and the correlated allele frequencies between populations, with values of K set from 1 to 8. The population information was incorporated into the analyses (LOCPRIOR model). Each run consisted of a burn-in period of 5^4^ steps followed by 10^6^ Monte Carlo Markov Chain (MCMC) replicates assuming an admixture model and correlated allele frequencies. K is the probable maximum population number that is assumed to represent and to contribute to the genotypes of sampled individuals. To check the consistency of the results between runs with the same K, eight replicates were run for each assumed K value. The approach suggested by Evanno et al. [46] was adopted to calculate the most likely value of K based on the second-order rate of change in the likelihood function with respect to K (ΔK). Once the number of genetic clusters was established, each individual was assigned to a cluster, and the overall membership of each sampled individual in the cluster was estimated.

The SilicoDArT and the SNP data were analyzed using DARTR [47]. Markers were scored “1” for presence, “0” for absence and “-” for failure to score. A distance matrix and a PCA graph were obtained using DARTR. The distance matrix was then converted to be used with the Darwin software.

### 4.6. Quality Analysis of Marker Data

The SNP and SilicoDArT results were tested for reproducibility (%), call rate (%), Polymorphism Information Content (PIC) and one ratio following Allam et al. [22].

## 5. Conclusions

This study allows us to deepen the cowpea genetic diversity knowledge of Portuguese and Mozambique landraces, recently stored in their germplasm banks and also still cultivated on-farm. Considering that on-farm conservation is a relevant strategy to maintain the evolutionary forces within and between components of the agricultural system [48], it is desirable to make efforts to safeguard this germplasm. Our results confirm that in Portugal and Mozambique cowpea, genetic variation has been maintained locally by farmers in some areas.

The diversity of cowpea landraces suggests the existence of a valuable gene pool for exploitation in future cross-country breeding programs. A large number of polymorphic markers obtained by SNP and SilicoDArT were identified and are now available as important and useful knowledge for genome studies.

## Figures and Tables

**Table 1:** Genetic diversity across twelve SSR loci. Na—number of alleles; Ne—effective number of alleles; Ho—observed heterozygosity; He—expected heterozygosity; PIC—Polymorphism Information Content.

Locus	Na	Ne	Ho	He	PIC
VM5	5	1.873	0.591	0.406	0.354
VM31	11	2.336	0.111	0.394	0.822
VM35	17	2.802	0.631	0.541	0.857
VM36	18	2.522	0.418	0.527	0.741
VM39	11	2.072	0.378	0.459	0.665
VuUGM05	26	3.439	0.199	0.462	0.834
VuUGM33	26	4.172	0.546	0.689	0.892
VuUGM71	27	4.588	0.682	0.710	0.851
CLM0634	4	1.781	0.053	0.282	0.403
CLM0721	16	1.975	0.100	0.424	0.618
CLM0767	7	1.807	0.003	0.290	0.401
CLM0792	15	1.947	0.187	0.348	0.645
Average	15.25	2.61	0.33	0.46	0.67

## Data Availability

All data are contained within the article and Supplementary Materials.

## Supplementary Materials

The following supporting information can be downloaded at: https://www.mdpi.com/article/10.3390/plants12040846/s1, Figure S1: Distribution of SilicoDArT and SNP marker data for several quality parameters, Figure S2: DeltaK, Table S1: Cowpea accessions used in this study, Table S2: Polymorphic SilicoDArT markers, Table S3: PIC values for SilicoDArT and SNP markers, Table S4: SNP markers, Table S5: Percentage of SNP types obtained in this study, Table S6: Genetic dissimilarity estimated through SSR markers, Table S7: Genetic dissimilarity estimated through SilicoDArT markers, Table S8: Genetic dissimilarity estimated through SNP markers, Table S9: SSR structure subpopulations and PCA axis values, Table S10: SilicoDArT structure subpopulations, Table S11: SNP structure subpopulations, Table S12: Cowpea primers.
