# Supplementary material for: Genetic Diversity and Population Structure of Cowpea (Vigna unguiculata (L.) Walp.) Landraces from Portugal and Mozambique
Source: Plants (Basel). 2023 Feb 13;12(4):846. doi: 10.3390/plants12040846 (PMC9963184; doi:10.3390/plants12040846)
Supplement: Supplementary file 1 [file plants-12-00846-s001.zip › Table S5 - Percentage of SNP types obtained in this study.pdf]

Table S5. Percentage of SNP types obtained in this study

Authors:

Joana B Guimarães et al

Correspondence:

catia.soares@iniav.pt

SNP Type	Number	Proportion	Percentage
G/A	1664	0.1506	15.06
C/T	1566	0.1417	14.17
T/C	1494	0.1352	13.52
A/G	1404	0.1271	12.71
T/A	934	0.0845	8.45
C/A	752	0.0681	6.81
A/T	680	0.0615	6.15
G/T	606	0.0548	5.48
G/C	516	0.0467	4.67
A/C	490	0.0443	4.43
T/G	484	0.0438	4.38
C/G	460	0.0416	4.16
**Total**	11050	1.0000	100.00
