# Supplementary figures and images for: Genetic Diversity and Population Structure of Cowpea (Vigna unguiculata (L.) Walp.) Landraces from Portugal and Mozambique
Source: Plants (Basel). 2023 Feb 13;12(4):846. doi: 10.3390/plants12040846 (PMC9963184; doi:10.3390/plants12040846)

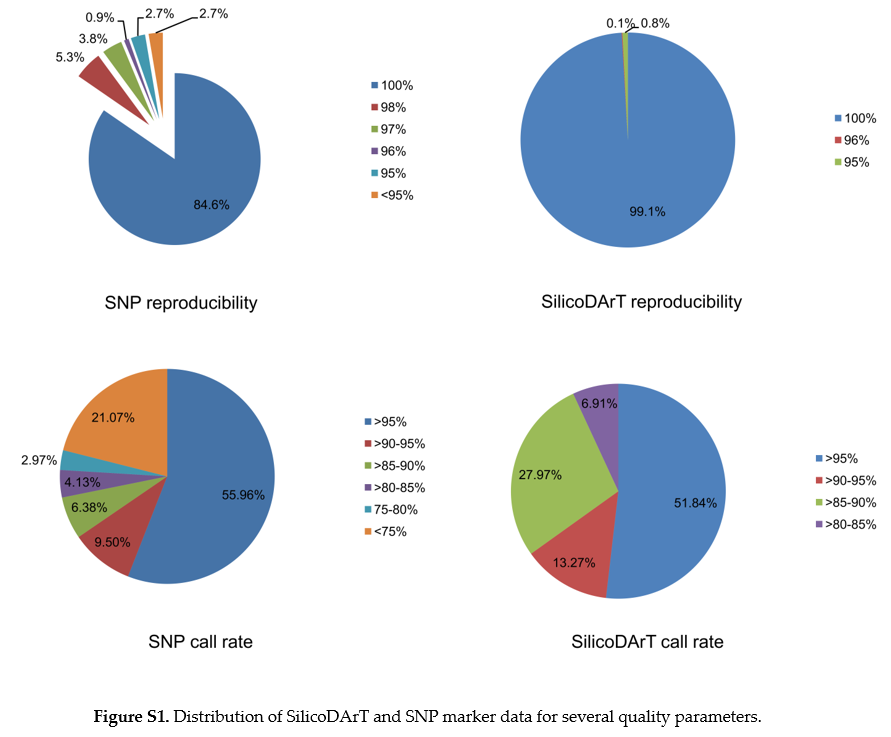

Supplement: Supplementary file 1 [file plants-12-00846-s001.zip › Figure S1 - Distribution of SilicoDArT and SNP markers data for several quality parameters.jpg.png]

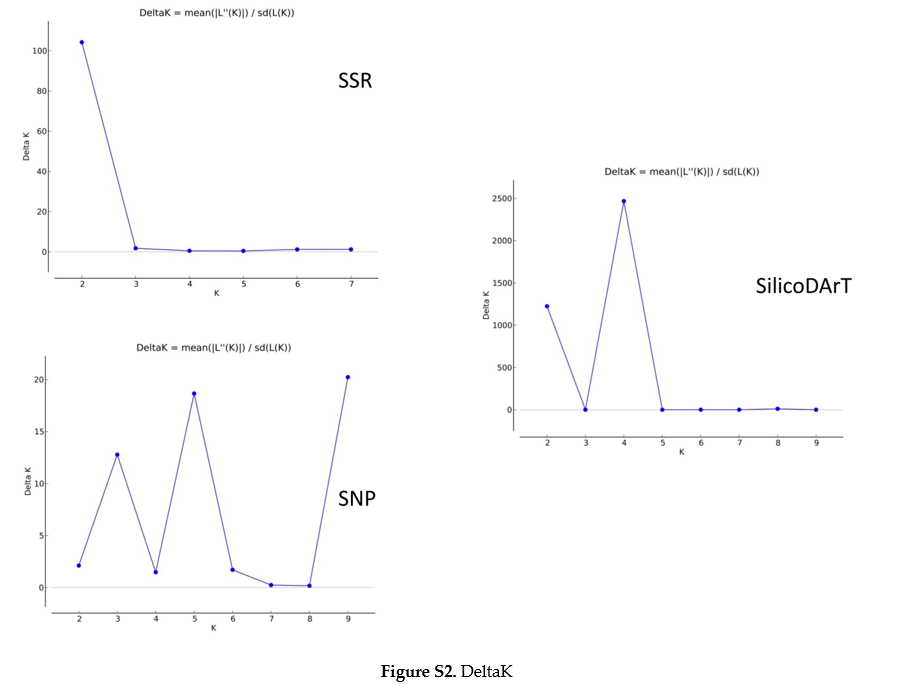

Supplement: Supplementary file 1 [file plants-12-00846-s001.zip › Figure S2 - DeltaK.jpg.png]
